# Assessing the role of inter-facility patient transfer in the spread of carbapenemase-producing *Enterobacteriaceae*: the case of France between 2012 and 2015

**DOI:** 10.1038/s41598-020-71212-6

**Published:** 2020-09-10

**Authors:** Narimane Nekkab, Pascal Crépey, Pascal Astagneau, Lulla Opatowski, Laura Temime

**Affiliations:** 1 Laboratoire MESuRS, Conservatoire National Des Arts Et Métiers, Paris, France; 2 Unité PACRI, Institut Pasteur, Conservatoire National Des Arts Et Métiers, Paris, France; 3 EHESP, REPERES (Recherche en pharmaco-épidémiologie et recours aux soins) – EA 7449, University Rennes, Rennes, France; 4 Centre régional de prévention Des Infections associées Aux Soins (CPias), Paris, France; 5 INSERM, Institut Pierre Louis D’Epidémiologie Et de Santé Publique, Sorbonne Université, 75013 Paris, France; 6 UMR 1181, «Biostatistics, Biomathematics, Pharmacoepidemiology and Infectious Diseases» (B2PHI), University Versailles Saint Quentin en Yvelines, Saint Quentin en Yvelines, France; 7 Pharmacoepidemiology and Infectious Diseases Unit, Institut Pasteur, Paris, France; 8 Inserm UMR 1181 (B2PHI), Paris, France

**Keywords:** Epidemiology, Statistics, Network topology

## Abstract

The spread of carbapenemase-producing *Enterobacteriaceae* (CPE) in healthcare settings is a major public health threat that has been associated with cross-border and local patient transfers between healthcare facilities. Since the impact of transfers on spread may vary, our study aimed to assess the contribution of a patient transfer network on CPE incidence and spread at a countrywide level, with a case study of France from 2012 to 2015. Our results suggest a transition in 2013 from a CPE epidemic sustained by internationally imported episodes to an epidemic sustained by local transmission events through patient transfers. Incident episodes tend to occur within close spatial distance of their potential infector. We also observe an increasing frequency of multiple spreading events, originating from a limited number of regional hubs. Consequently, coordinated prevention and infection control strategies should focus on transfers of carriers of CPE to reduce regional and inter-regional transmission.

## Introduction

Within the *Enterobacterales* order, the emergence and increasing number of carbapenemase-producers among the *Enterobacteriaceae* family pose a major threat to healthcare systems and jeopardizes patient safety^1–3^. An alarming report by the European Centre for Disease Control (ECDC) in 2015 described regional and inter-regional spread of carbapenemase-producing *Enterobacteriaceae* (CPE) including four countries in which CPE has become endemic^4^. CPE spread and subsequent outbreaks have been linked to transfers of patients between healthcare facilities within countries and across national borders^5–7^. While new antibiotics to replace carbapenems and to treat CPE infections such as cefiderocol, ceftazidime-avibactam, and carbapenem-beta-lactamase inhibitor combinations have recently become available^8^, controlling the spread of CPE across healthcare systems with other measures is essential. This requires better understanding of the impact of transfer patterns on the spread of nosocomial pathogens.

Over recent years, several studies have correlated measures of healthcare facility connectivity in healthcare networks of patient transfers to CPE incidence, underlining the importance of coordinated control measures at the regional scale^9–13^. However, many questions have been left unanswered. In particular, despite studies documenting the transmission chains of cross-border transfer of CPE strains and healthcare facility outbreaks, the overall contribution of inter-healthcare facility transfers on spatial dispersal of CPE over time has not yet been assessed at a national-level in any country in particular.

In this study, we aimed to investigate the contribution of patient transfers on the spread of CPE in France and, in addition to the observations that have been made over the past few years, to further describe the CPE epidemic. Indeed, on the one hand, an increasing number of OXA-48 cases (class D beta-lactamases with oxacillinase activity) coming into France from cross-border transfers have been reported in recent years^4–6,14,15^. On the other hand, despite updated national guidelines and strategies, from the 2013 to the 2014–2015 period France advanced from an epidemiological stage-3 of regional spread to a stage-4 of inter-regional spread of CPE^16^.

In order to assess the extent to which patient transfers may have contributed to the transition from regional to inter-regional spread of CPE in France, we exploited exhaustive datasets of inter-hospital patient transfer network in France and all reported CPE episodes to empirically test the contribution of the network on CPE episode incidence. For the 2012 to 2015 period, we evaluated the number of CPE episodes potentially linked to patient transfers in France and determined whether the transfer network supported the CPE incidence data.

## Results

### Patient transfers and CPE episodes

A total of 2.3 million patients with a total of one million direct transfers were recorded in France in 2014^17^. The network of patient transfers was comprised of 2063 healthcare facilities (including long-term care facilities) which had at least one transfer during the year (excluding the overseas counties). Aggregation to the county level created a network of 96 administrative counties spanning continental France with 3,482 connections between them. A total of 208,312 transfers occurred between facilities of different counties. A mean of 60 patients were transferred between counties with a maximum of 7,145 patient transfers within one connection.

**Figure 1:**
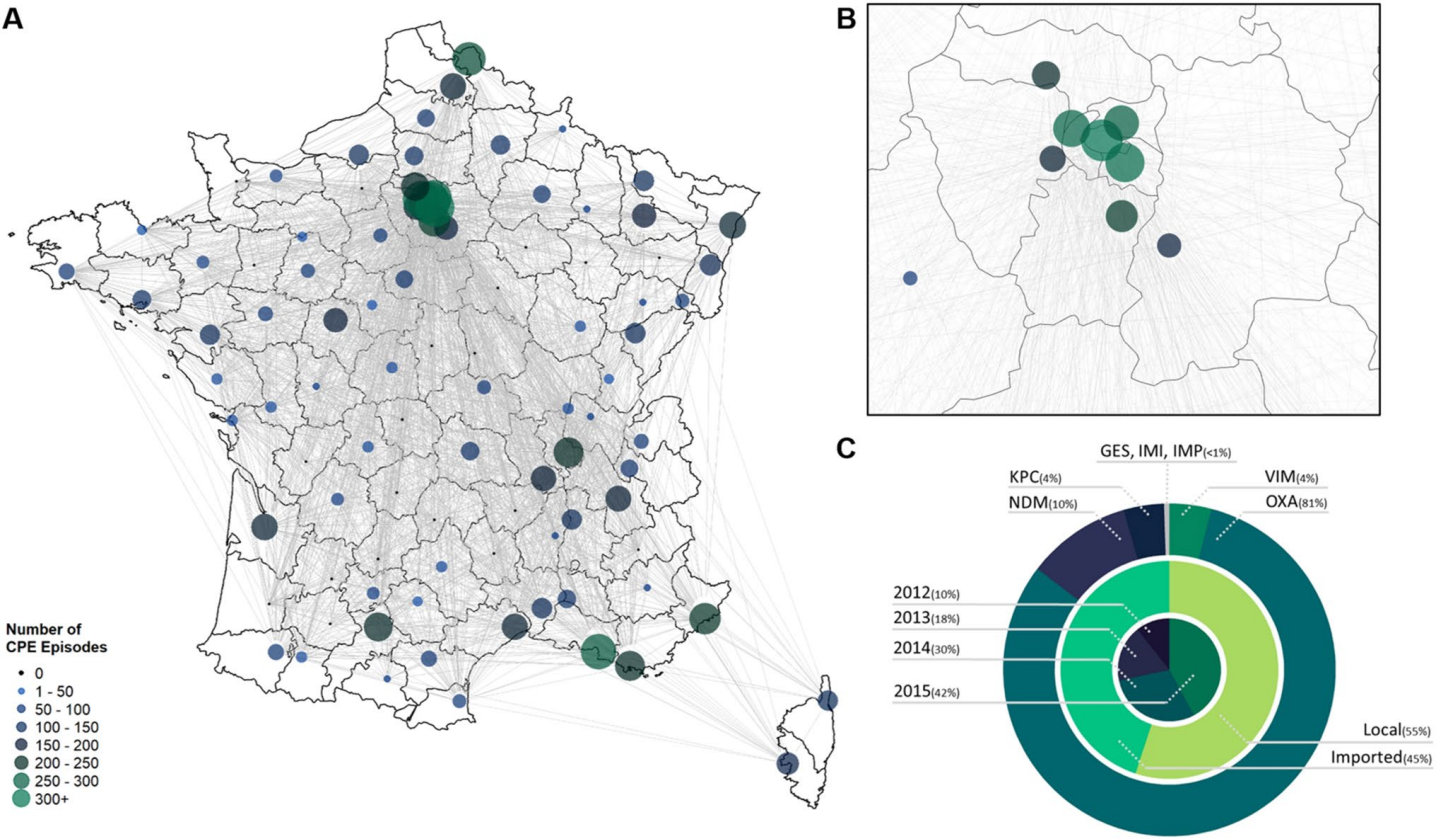
Network of patient transfers between French counties and incident CPE episodes reported between 2012 and 2015. (**A**) The county network is comprised of 96 counties linked together by over 3,000 connections (grey lines). Patient transfer links were centralized to the county seat. For each county, the cumulative number of incident CPE episodes reported in healthcare facilities of the county from 2012 to 2015 is depicted by a circle. (**B**) A zoomed-in view of the Ile-de-France region. (**C**) A multi-level pie chart showing the proportion of incident CPE episodes for each year, along with their importation status and mechanisms of resistance.

A total of 2,277 CPE episodes occurred in French healthcare facilities from January 2012 to December 2015. Among these episodes, all three classes of carbapenemases (A, B and D^18^) were reported, with 81% being of class D OXA-enzyme producing *Enterobacteriaceae*. The number of CPE episodes increased almost four-fold during the four-year time span reaching a total of 956 episodes in 2015. The proportion of episodes not linked to importation from cross-border transfer increased over time from 48% in 2012 to 60% in 2015. Overall, 1,253 episodes were classified as non-imported. Episode localization was restricted to the county level. Figure 1 shows the total number of CPE episodes reported in each county over the 2012–2015 period and links between counties based on the 2014 patient transfer network structure.

### Does the transfer network support CPE incidence?

Our baseline results showed that 90% (or 1,125) of the 1,253 non-imported CPE episodes reported between 2012 and 2015 could be linked to a “potential infector” i.e. an episode with the same reported mechanism of resistance occurring 21 to 28 days before the incident episode (Table 1). Out of the 1,125 pairwise episodes, 87.6% were OXA-48 strains. Observed network distances between the incident episodes and their potential infectors were significantly shorter than the network distances between the same incident episodes and their potential infectors determined from random permutations of the county of the incident episode (Wilcoxon paired rank sum test, *p* value = 2.71 × 10^−27^). This suggested that the transfer network did indeed support CPE spread. In addition, significantly more potential infectors were identified in the same country as the incident episode than expected by chance (Supplement 1).

**Table 1:** Characteristics of 2,277 CPE episodes in France from 2012 to 2015.

Year	2012–2015	2012	2013	2014	2015
Total episodes	2,277	242	405	672	958
Imported episodes	1,024	125	201	311	387
Non-imported episodes (%)	1,253 (55%)	117 (48%)	204 (50%)	361 (54%)	571 (60%)
Non-imported episodes with potential infector (%)	1,125 (90%)	97 (83%)	177 (87%)	307 (85%)	498 (87%)
NSP distance of observed data mean [95% CI]	5.61 [5.35–5.87]	7.34 [6.41–8.27]	6.15 [5.50–6.81]	5.92 [5.43–6.40]	4.86 [4.49–5.24]
NSP distance of permutations mean [95% CI]	5.89 [5.65–6.12]	7.24 [6.46–8.02]	6.32 [5.69–6.95]	6.13 [5.69–6.57]	5.35 [5.01–5.68]
*p* value*	2.71 × 10^−27^	0.14	0.001	9.78 × 10^−7^	7.17 × 10^−20^

Potential infectors were identified for a total of 1,125 episodes over the entire 2012 to 2015 period. In year-specific analyses, CPE data only contained episodes occurring from January 1st to December 31st; therefore, the search for potential infectors was restricted to this period. As a result, 128 potential infectors identified in the global analysis were not included in the stratified analyses.

*NSP* network-supported path.

*Wilcoxon paired rank sum test *p* value comparing NSP distances between observed and permuted data.

**Figure 2:**
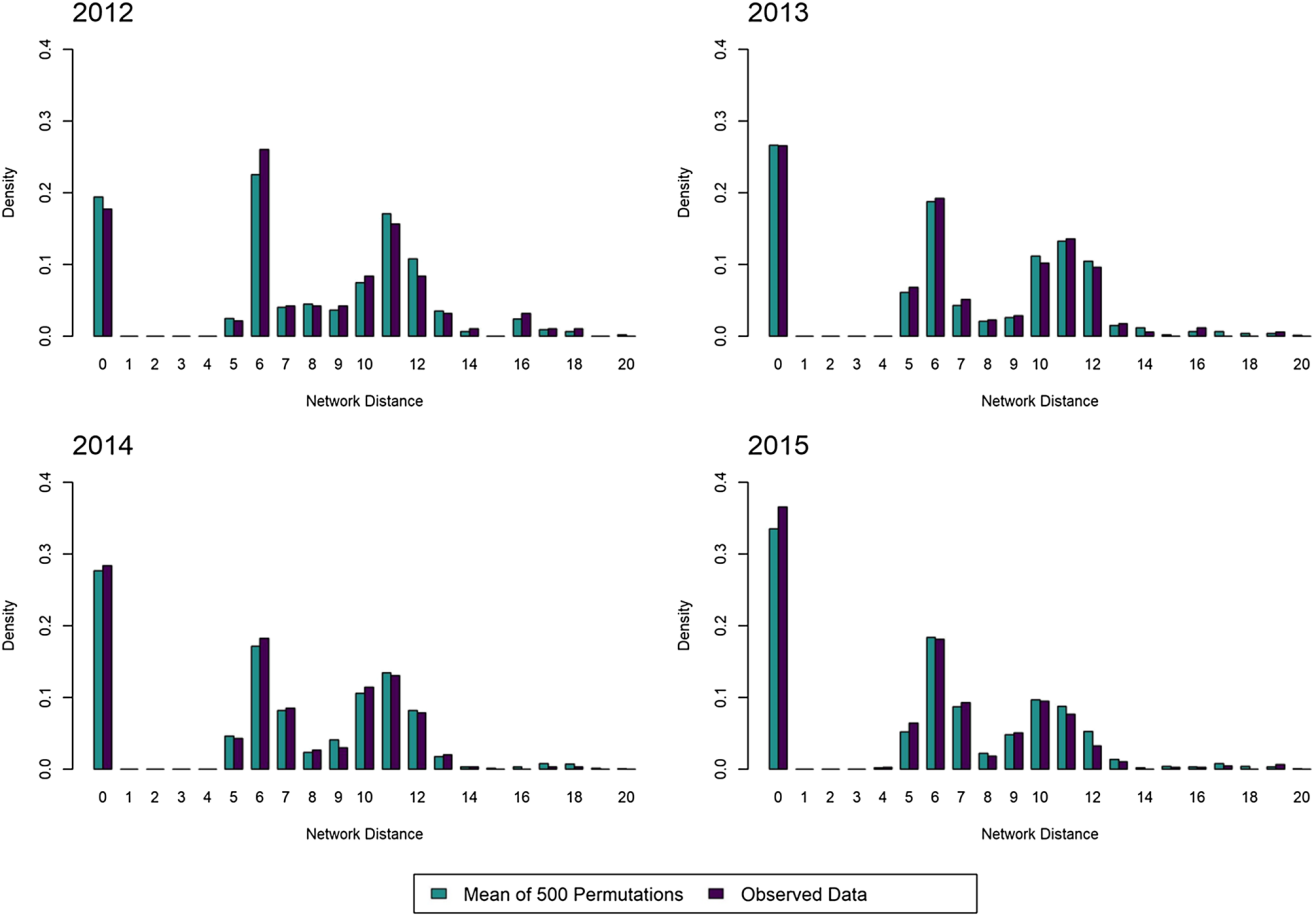
Comparison of the distributions of network distances for observed vs. permutated data, 2012–2015. For each year, the density distribution of distances between incident episodes and their potential infector in the observed data is shown; as well as the density distribution of the mean distances computed based on 500 random permutations of the data, illustrating the distribution expected under the null hypothesis of independence between patient transfers and CPE incidence. Distances of zero correspond to linked episodes occurring in the same county. Distances of one correspond to a range of distances between zero and one; the same applies to distances of 2–20.

When these results were stratified by year, a potential infector was identified over the county network for 83% (in 2012) to 87% (in 2015) of all reported non-imported episodes (Table 1). Observed network distances were significantly shorter than the distances of the permutations of the data for 2013, 2014 and 2015 episodes (Wilcoxon paired rank sum test, *p* value = 0.001, 9.78 × 10^−7^, 7.17 × 10^−20^ respectively). Conversely, in 2012, network distances did not differ significantly (Wilcoxon paired rank sum test, *p* value = 0.14). Figure 2 compares the density distribution of the network distance for each year.

### Intra- and inter-county spread

**Figure 3:**
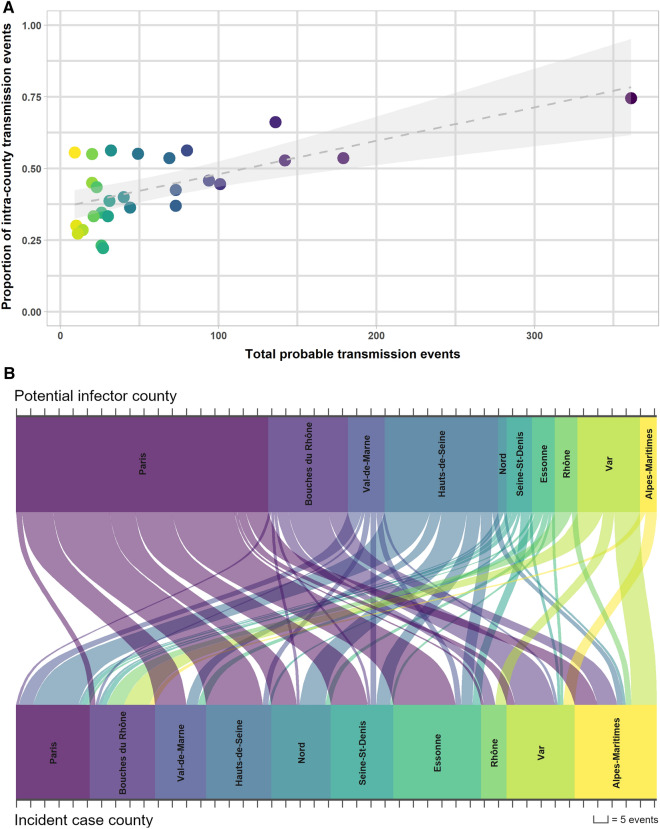
Inter and intra-county probable transmission events, 2013–2015. (**A**) For each county, the total number of pairwise episodes or “probable transmission events” for the 2013–2015 period were compared to the proportion of these events that occurred within the same county. A simple linear regression model with 95% confidence intervals is shown. Colors represent individual counties from largest number of total events (purple) to least (yellow). (**B**) The Senkey diagram shows the number of linked events between the counties of the potential infector and the counties of the incident episodes. To reduce noise, we limited our observations to only the inter-county links of ten counties with the highest number of total probable transmission events.

For the 2013 to 2015 network-supported period, we examined the observed pairwise county links or “probable transmission events.” The number of counties implicated in these events doubled from 10 counties in 2013 to 20 counties in 2015. We observed that as an increasing number of events in a county occurred, the proportion of both episodes occurring within the same county increased (Fig. 3A). During this period, an average of 43.4% of CPE transmission events occurred within counties. On the other hand, the proportion of these intra-county probable transmission events decreased over time from 55.3% in 2013 to 44.5% in 2015 suggesting that CPE inter-county spread played an increasingly more important role.

Concerning these inter-county probable transmission events, we observed that Paris played a significant role as a probable source of CPE transmission events across the territory implicating most of the other counties (Fig. 3B). Other important potential infector counties such Bouches-du-Rhône, Hauts-de-Seine and the Var led to less dispersion across the network as compared to Paris implicating mainly three other neighboring counties. In addition, the majority of inter-county probable events were within close spatial distance of one another and the average spatial distance between them reduced over time (from 225 km in 2013 to 163 km in 2015) (Supplement 2).

### Multiple spreading events

**Figure 4:**
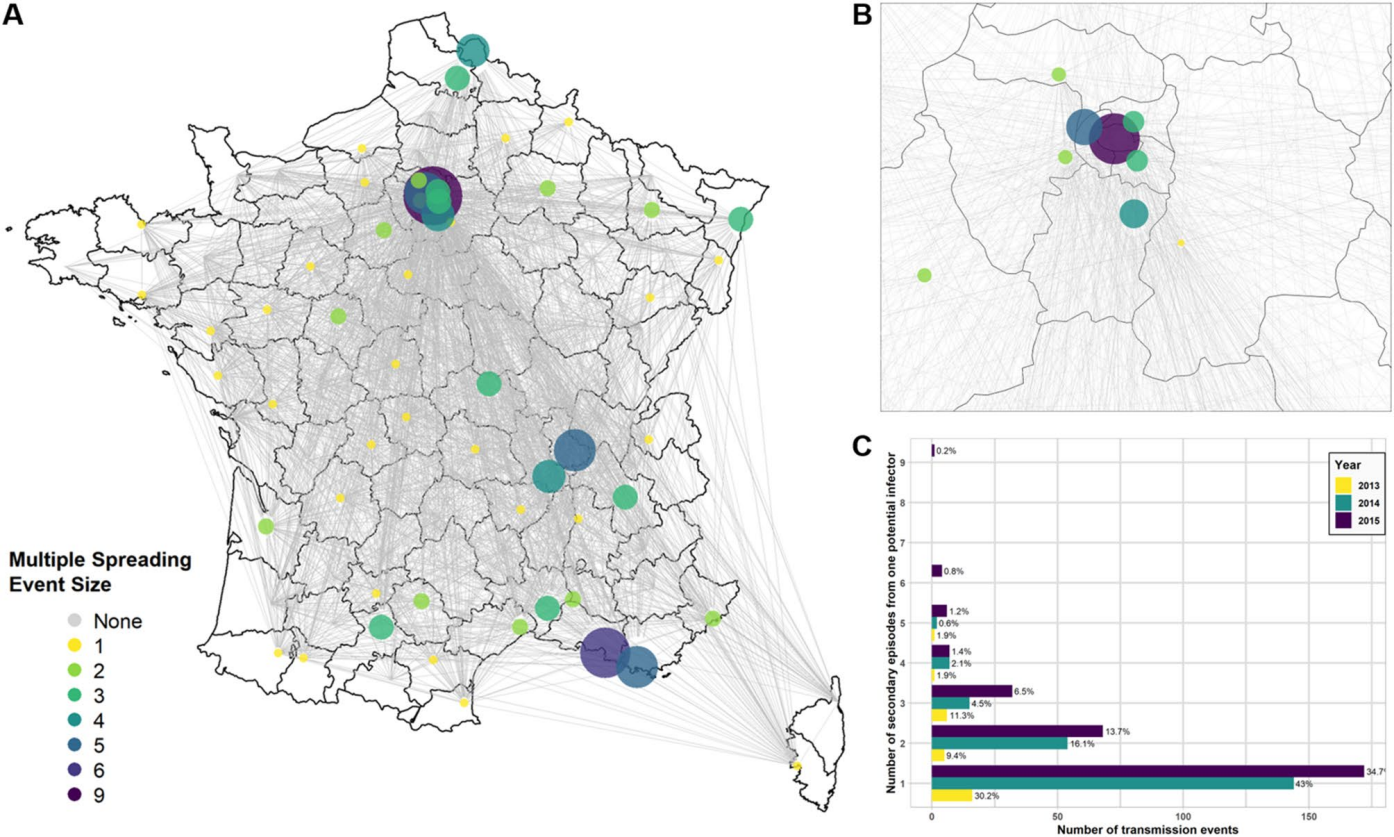
Multiple spreading events and number of potentially associated secondary incident episodes, 2013–2015. (**A**) The county network mapping the size or number of linked secondary events to one potential infector episode from 2013 to 2015. (**B**) A zoomed-in view of Ile-de-France counties including Paris (purple). (**C**) The distribution of the total number of transmission events and the size of the multiple spreading events with proportions per year.

Since potential infector selection was performed independently for each incident episode, incident episodes could share the same potential infector. Multiple spreading events were thus defined as events in which more than one incident episode shared the same potential infector. The size of the event was defined as the largest associated number of secondary events in each potential infector county during the study period. In Fig. 4A, we can observe that the largest size of these events ranged from 1 to 9 potentially associated secondary episodes (Fig. 4A). The proportion of multiple spreading events out of all possible transmission events varied from 57.0% to 69.8% with no apparent trend over time (Fig. 4C). However, we did observe that increasingly larger probable multiple spreading events occurred in 2015 with two counties spreading CPE to six other counties each and the Parisian county spreading CPE to nine other counties (Fig. 4B).

In order to assess if a large outbreak episode was responsible for multiple spreading events to other counties, we ranked all potential infector episodes by the number of reported cases associated with the episode and the size of the spreading event. We did not find evidence of an association between the number of cases of the potential infector episode and the size of the multiple spreading events suggesting that CPE outbreaks may have not contributed to county-level spread mediated by patient transfers (Kendall’s rank correlation tau with averaged ties, *p* value = 0.05, 0.14, and 0.39 for 2013, 2014, and 2015 data respectively).

### Sensitivity analysis

**Figure 5:**
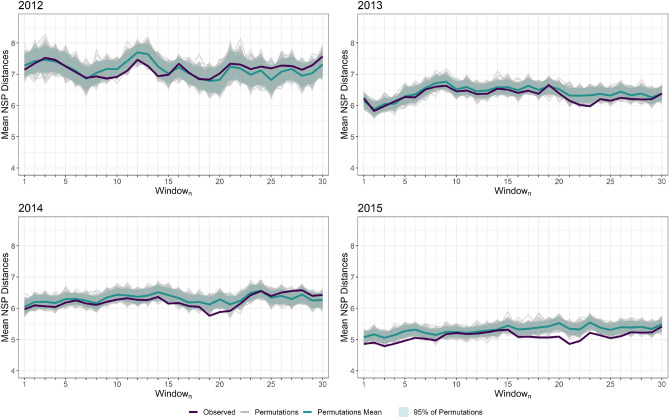
Sensitivity analysis on the impact of the time window chosen to look for candidate transmitters: mean network distances between incident episodes and their closest potential infectors obtained for sliding 1-week time windows, 2012–2015. For each year, the mean network distance is plotted as a function of the first day of the 1-week time window, Window_n_, for observed data and permuted data. For the permutations, 95% bands are also provided.

We conducted a sensitivity analysis on the time window used to select the potential infector by moving a one-week window from 1 to 23 days prior the incident episode (Fig. 5, Supplement 1). An association between patient transfers and incident episodes from 2013 to 2015 was most significant when potential infectors were identified 20 to 30 days before the incident episode date, validating our choice of a 21–28 days baseline window. *p* values of these results can be found in Supplement 3.

## Discussion

To the best of our knowledge, our study is the first to examine the impact of patient transfers on CPE transmission at a country-wide level. We were able to adapt a previously proposed statistical methodology to assess whether patient transfers were associated with CPE dissemination in France over a 4-year period^19^. We calculated the network distances between likely infector episodes (both imported and non-imported) and non-imported incident episodes of CPE by accounting for the number of patient transfers. By showing that the network distances in the observed data were significantly shorter than the distances we would have expected if CPE episodes were to occur at random across the counties, our study suggested that there was an association between the French patient transfer patterns at the county level that contributed to the spread of CPE.

In order to select the most probable potential infector episode, we estimated from published reports that multi-healthcare facility CPE outbreaks in France last approximately three weeks and that detection of cases between different healthcare facilities occurred three to four weeks after detection in the index healthcare facility^20^. Since one episode may lead to a chain of transmission events in other healthcare facilities and in our case multiple secondary episodes, we selected a cut-off window for potential infector selection. We chose a baseline window of 7 days spanning from 21 to 28 days prior to the date of the incident episode for potential infector identification to reduce the bias of linking incident episodes to other secondary episodes (e.g. to link episodes within the appropriate serial interval rather than “siblings” from the same infector episode). This baseline window was consistent with French epidemiological data on observed delays before detection of CPE cases within a hospital following transfer of colonized patients^21,22^. It was also supported by the sensitivity analysis results where we observed that, among all windows, it provided one of the lowest observed mean distance as well as one the largest difference from the permutations in 2015 (Supplement 3). It is important to note that other windows also gave significantly shorter distances around the baseline especially in 2014 and 2015. These results are a feature of the French healthcare system and may vary from one healthcare system to another since they rely on how CPE episodes are reported to surveillance authorities and the patient transfer protocols. Our methods however, can be applied elsewhere and may serve as an important tool for identifying high-risk periods of hospital-to-hospital CPE transmission and when control strategies could be most pertinent.

Our results suggest that the dynamics of CPE transmission in France have changed over time. Between 2012 and 2015, we were able to show evidence using network distances to evaluate the changing dynamics of CPE spread; evidence that supports potential CPE spatial spread through the carriage of CPE by transferred patients between French counties. CPE episodes from 2012 were not supported by the transfer network and may be better supported by other sources of transmission such as stays in a foreign country, direct cross-border transfers, antimicrobial consumption, or long carriage duration from previous hospitalisations. On the other hand, the increasing proportion of non-imported episodes after 2012 and statistically significant associations between the transfer network and CPE incidence suggests a transition in 2013 from an epidemic previously sustained by importation to an epidemic sustained by local transmission events. Mounting evidence for local spread through transfers emerged in 2014 followed by the strongest evidence for network-supported CPE transmission in 2015. These results suggest that between 2013 and 2014 there was a growing contribution of regional and inter-regional transfers in the spread of CPE in France which is in concordance with reports by the ECDC^16^. The transition of the CPE epidemic from importation to local spread and from regional to inter-regional spread may have also occurred in other European countries^4,7^. Similar studies in other context are needed to explain if these associations are unique to France or are part of a larger phenomenon in the international spread of CPE.

While the hub county of Paris may be responsible for most imported CPE episodes in France and also the dispersion of a large number of probable transmission events across the network, most of these events were predicted to occur within the county itself. We may find that at a finer spatial resolution—the hospital-level—in which CPE transmission may be more relevant to understand these spatio-temporal dynamics—that only a small proportion of hospitals actually contribute to spread further away while the vast majority of infections spread more locally as observed in England^23^. We may also over-estimate the probable risk in inter-county transmission events due to hospitals sharing patients along the county borders, which is also supported by the observed shorter spatial distances between these counties over time. Despite these limitations, we observed that the number of counties implicated in transmission events doubled over the three-year period with increasing network-supported transmission. Our results suggest the importance of developing two different strategies to combat CPE spread: an intra-county strategy focusing on controlling local spread among frequent community transfers and readmissions and an inter-country strategy targeting less common but potentially riskier inter-county transfers.

Due to no observed association between the number of cases per potential infector episode and the number of secondary episodes, we were not able to show evidence of poor control of healthcare facility CPE outbreaks once health authorities identified and reported a chain of transmission among cases. On one hand this may suggest that control measures have prevented large healthcare facility outbreaks from causing multi-county outbreaks during the 2014–2015 period; on the other hand, most reports are single-case episodes located within close spatial distances, which might suggest a failure of surveillance authorities in identifying single-cases as part of the same chain of transmission of other reported episodes. In addition, CPE carriers are likely to be discharged in the community which can lead to ongoing transmission, non-identification and poor control of any potential spread. Guidelines for screening and controlling CPE should include those epidemiological changes and be revised accordingly.

In the context of our study, we were subject to a certain number of limitations that are important to address. One major assumption that our work relied on was that the patient transfer patterns in 2014 did not vary from other years and that they could support the incidence of CPE episodes in 2012, 2013, and 2015. These assumptions relied on previous work suggesting that patient transfer patterns do not change significantly over time^17^. Since the healthcare facilities were aggregated to the county-level, any changes in the number of facilities in which patients were transferred (e.g. local hospital mergers) would not have impacted the results; however, any changes in the number of patients transferred between counties could have potentially impacted the calculation of network distances and the selection of potential infectors. However, since the majority of the incident episodes and potential infectors occurred in the same county (with a distance of zero) or within short spatial distances, only a very significant increase or decrease in the number of patient transfers could potentially modify our results, which may not have been likely.

The patient transfer network included transfers from hospitals, healthcare centres, and long-term care centres including some nursing homes; however, our dataset was not fully exhaustive of all nursing homes. Including all nursing home transfers may not have changed the main results of our study since patient transfers were aggregated to the county level and the number of transfers from these facilities may have been small. In addition, the network did not include patient transfers directly from the community but rather direct healthcare facility-to-facility transfers. Including transfers from the community may be important in identifying any potential transmission outside healthcare facilities; however, our study aimed to link only CPE episodes detected and reported in the healthcare setting. In addition, CPE episodes in the community are rarely detected and reported; however, they should be considered in future research and are imperative to understanding the full scope of CPE spread.

Although we did not evaluate the role of other CPE risk factors such as previous hospitalizations or antimicrobial consumption, these factors may have also contributed to the emergence and spread of CPE within the territory; however, they may have played an increasingly less significant role over time. International importation could have contributed to almost half of the spread of CPE in the country. These results are consistent with the outbreak descriptions in the literature in which both imported and non-imported cases led to secondary cases of CPE in different healthcare facilities. The mix of patient transfers along with heterogeneity in infection control policies across different types of healthcare facilities, high antimicrobial consumption, and limited implementation of specific strategies to control CPE such as routine screening for CPE carriage among transfers may have led to poor control of CPE and in consequence, dissemination over time^24^.

Although CPE reporting is obligatory in France, not all healthcare facilities may detect and report episodes of CPE in the same fashion. Cases of asymptomatic carriage of CPE may also go undetected by the surveillance system. As a result, such studies relying on only surveillance data may be subject to bias and are limited to only explaining the incidence of notified CPE episodes. However, despite the limitations of the data, we were still able to show that patient transfers may indeed be an important mechanism of CPE spread. As a result, taking precautions regarding at-risk patient transfers (i.e. in large highly connected metropoles that may harbour multiple spreading events) even when CPE carriage status is unknown at both the local hospital level and at the regional level could help reduce CPE transmission. Future modelling studies are needed to assess the impact of combining different prevention and control strategies on CPE spread.

In conclusion, our results suggest that since 2013, patient transfers in France have increasingly contributed to the epidemiological transition of CPE dynamics from regional to inter-regional spread sustained by an increasing number of local spreading events. Systematic screening of at-risk patients, such as healthcare facility contacts of patients transferred from healthcare facilities with previous or current patients infected with CPE is crucial in identifying carriers to contain inter-healthcare facility transmission. These efforts rely on regional coordination of control measures targeting patient transfers, especially that of healthcare facility centres that play an important role in connecting the patient transfer network^17^.

## Methods

### CPE episodes data

Surveillance data of 2,277 CPE episodes occurring in continental France from January 2012 to December 2015 were used in the analysis. Data were collected by Public Health France through the HAI-EWRS active surveillance system. The French independent regulatory body (Commission nationale de l’informatique et des libertés (CNIL)) permitted the use of the data.

CPE episodes were defined as a case or group of cases infected with the same strain of CPE known to have been in contact with one another and identified by authorities during the outbreak investigation. Episodes were described by the number of individual cases involved in the chain of transmission, county of occurrence (“département” in French; an administrative division between the regions and communes), date (date of the first detected case), mechanism(s) of CPE resistance, bacterial species, and site of infection or colonization if known. Episodes were classified as “imported” if the index case of the chain of transmission was initially infected or colonized in a foreign country. We assumed that for episodes in which there were multiple cases, the cases all occurred in the same county.

### Patient transfer network

The network of patient transfers between hospitals and healthcare centres (including long-term care services such as postoperative and rehabilitation care) in 2014 was built and described in detail in a previous study by the authors^17^. Patient transfer data was collected from the national hospital discharge database, a comprehensive medico-administrative database of patient discharge summaries. Only direct hospital-to-hospital or medical ward transfers were considered. The hospital network was transformed into an adjacency matrix of nodes representing administrative counties, with the edges representing the connections between counties. The county network edge weights were given as the sum of the number of hospital transfers between counties for the entire year of 2014. As shown in the previous study on the network, the number of patient transfers remained stable during the year. Therefore, we assumed that the county-level network structure did not change significantly from 2012 to 2015 and used to the number of inter-county transfers in 2014 network for the entire study period^17^.

Aggregation of healthcare facility data to the county level was necessary. Due to a lack of information regarding healthcare facilities in which the CPE episodes were reported, we relied solely on the county of the episodes. We assumed that transfer rates between healthcare facilities of the same county were homogenous and had sufficient heterogeneity in the transfer rates between counties to proceed with the analysis.

### Potential infector identification

In the methodology proposed by Obadia and colleagues^25^, the authors used a hospital network of patients and healthcare workers contacts to identify, for incident colonization episodes of *Staphylococcus aureus*, the potential infectors that were best supported by the network in terms of path distance. The distribution of observed path distances between incident cases and their closest potential infector was compared to that obtained using randomly distributed colonization data over the same network.

Here, rather than a contact network, we used the county network of patient transfers to identify the most likely potential infector for CPE incident episodes. We assumed that transmission could have occurred equally among episodes with colonised or infected cases or both and among all genera of *Enterobacteriaceae*. Each non-imported incident CPE episode was investigated using the following algorithm:

1. All episodes involving CPE with a shared mechanism(s) of resistance that occurred within a specific time window prior to the incident non-imported episode in any network county were considered “candidate transmitters” (see Table 2). We assumed that non-specific CPE strains such as KPC, NDM, or VIM were possible candidates transmitters to either the same non-specific strain or a specific strain due to the uncertainty in the data.
2. All network distances between the incident episode county and all candidate transmitter counties were compiled from a matrix of total annual transfers of patients between each county
3. For a given incident episode, the candidate transmitter with the shortest network distance length between its county and the incident episode county was considered as the most likely potential infector

**Table 2:** Shared mechanisms of resistance.

Incidence episode mechanism(s)	Candidate mechanism(s)
OXA-48	OXA-48
OXA-48-like	OXA-48-like
OXA-181	OXA-181
OXA-204	OXA-204
OXA-244	OXA-244
KPC	KPC or any specific KPC
KPC-2	KPC or KPC-2
KPC-3	KPC or KPC-3
NDM	NDM or any specific NDM
NDM-1	NDM or NDM-1
NDM-4	NDM or NDM-4
NDM-5	NDM or NDM-5
NDM-7	NDM or NDM-7
VIM	VIM or any specific VIM
VIM-1	VIM or VIM-1
VIM-2	VIM or VIM-2

Therefore, we defined “potential infectors” as the episodes with the shortest network path away from the incident episode among all “candidate transmitters.” We defined “candidate transmitters” as all episodes occurring *n* number of days before each incident episode who share the same carbapenem mechanism of resistance. Therefore, we defined these pairwise network distances or network-supported path (NSP) distances as the distance between the incident episode’s county and the county of its’ potential infector episode.

### Statistical methods

In order to statistically assess the potential impact of patient transfers on CPE spread, the distribution of pairwise NSP distances between each non-imported CPE episode and its potential infector was compared to the distribution expected under the null hypothesis of independence between CPE transmission and the county network of hospital transfers. Expected network distances under the null hypothesis were determined using a random permutation of the counties of all episodes (see section “Example of potential infector selection”). Five-hundred permutations (enough to ensure stability of results) were generated and the algorithm of potential infector selection followed for each permutation. Each incident episode and its new potential infector network distances from randomly permuted data were averaged, producing a distribution of network distances expected under the null hypothesis. For observations over the entire 2012 to 2015 period and for each year independently, the observed distribution was compared to the distribution of the permutated network distances using a paired Wilcoxon signed-rank test.

### Choice of a time window for candidate transmitter selection

Understanding the length of time it takes for CPE colonization or infection to be detected in one hospital after its contamination via patient transfers from another hospital is essential in being able to appropriately link CPE episodes. The estimation of this delay time relies on data from outbreak investigations.

The median duration of CPE outbreaks in French hospitals from 2004 to 2012 was estimated at 22 days^20^. In addition, a few studies have reported the delay time in the detection of CPE colonization or infection between healthcare facilities as a result of local patient transfers. In a multi-hospital outbreak of carbapenemase-producing *Klebsiella pneumoniae* (KPC), two patient contacts were transferred and detected positive in two other hospital facilities 15 and 29 days after the detection of the hospital index cases^21^. In another KPC outbreak, following patient transfers out of the hospital in which the outbreak originated, KPC colonization was detected in two other hospitals respectively 19 and 25 days after detection of the index case in the original hospital^22^.

Based on this data, for this study, we chose to look for potential infectors of incident CPE episodes in a 1-week time window ranging from 21 to 28 days (*W*_*[21,28*]_) before the incident episode *E*_*i*_. A sensitivity analysis was conducted to compare this baseline window to a sliding 1-week window starting from *W*_*[1,8]*_ to *W*_*[30,37]*_ before the notification of *E*_*i*_.

### Distance computation in the weighted county network

In order to identify the closest potential infectors over the weighted county network, we first converted the edge weights *w*_*ij*_ to annual transfer rates *t*_*ij*_ by dividing the total number *w*_*ij*_ of transfers from county *i* to county *j* by the sum of all patient stays in the origin county *i*:

$${t}_{ij}=\frac{{w}_{ij}}{{t}_{i}}$$

We then defined as the distance from county *i* to *j* as the negative log of the transfer rate *t*_*ij*_:

$${d}_{ij}=-\log({t}_{ij})$$

Shortest path distances were computed using Dijkstra’s algorithm with the R package “igraph”^26^.

### Example of potential infector selection

Here we show a hypothetical case to demonstrate how potential infectors were identified given hypothetical data (Table 3). For a given incident episode *E*_*i*_ during a given one-week sliding window, *W*_*[t-n,t-m]*_, in which *t* corresponds to the *E*_*i*_ date, *n* corresponds to the first day of the sliding window preceding the date of *E*_*i*_ and *m* corresponds to *n* + 7 days preceding *E*_*i*_, five CPE episodes *E*_*ae*_ occurred in five different counties. Among episodes that shared the same mechanism of resistance, the candidate transmitters *j*, *E*_*d*_ and *E*_*e*_, the shortest network path distance in the observed data was between *E*_*i*d_ (*d*_*id*_ = 1 < *d*_*ie*_ = 2). Therefore, in the observed data, the most likely potential infector of *E*_*i*_ was identified as *E*_*d*_ with a network distance equal to 1. The counties of *E*_*ae*_ were permutated through sampling without replacement 500 times and the potential infectors were identified. In permutation_1_, *E*_*e*_ was identified as the potential infector given that *d*_*id*_ = 4 > *d*_*ie*_ = 3 and, in permutation_500_ *E*_*d*_ was identified as the potential infector (*d*_*id*_ = 2 < *d*_*ie*_ = 3). The distribution of the observed network distances was compared to the mean of the network distances of the 500 permutations for each non-imported episode *E*_*i*_ for significance using a Wilcoxon paired rank sum test.

**Table 3:**
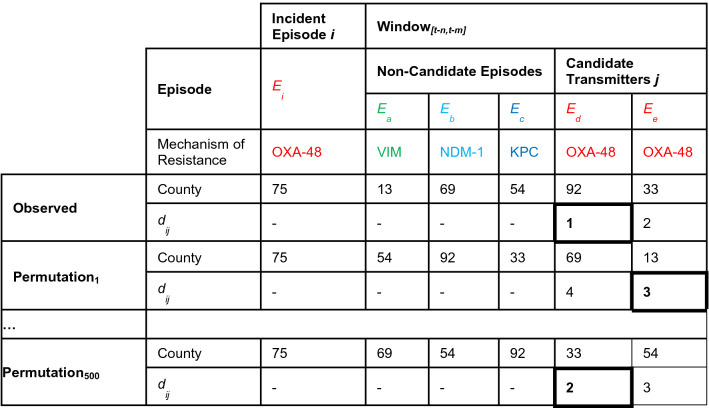
Example of potential infector selection in the observed data and permutations.

## Supplementary information

Supplementary Information.

## Data Availability

Aggregated data from figures can be made available upon request. The datasets analysed during the current study cannot be made publicly available because we do not legally own the data and public availability would compromise the privacy of the hospitals and the patients. The PMSI database of patient transfers is owned by the French Agence Technique de l'Information sur l'Hospitalisation (ATIH) and the CPE case data is owned by Santé Publique France. Access for the datasets requires meeting the criteria determined by the Commission nationale de l’informatique et des libertés (CNIL). For those wishing to have access to the databases, the request can be sent to demande_base@atih.sante.fr and more information on the ATIH can be found on their website www.atih.sante.fr.
