# Supplementary material for: Assessing the role of inter-facility patient transfer in the spread of carbapenemase-producing Enterobacteriaceae: the case of France between 2012 and 2015
Source: Sci Rep. 2020 Sep 10;10:14910. doi: 10.1038/s41598-020-71212-6 (PMC7483561; doi:10.1038/s41598-020-71212-6)
Supplement: Supplementary file 1 — Supplementary Information. [file 41598_2020_71212_MOESM1_ESM.pdf]

Assessing the role of inter-facility patient transfer in the spread of carbapenemase-producing *Enterobacteriaceae*: the case of France between 2012 and 2015

Narimane Nekkab[1,2,3], Pascal Crépey[3], Pascal Astagneau[4,5], Lulla Opatowski[6,7,8], Laura Temime[1,2]

Affiliations
[1] Laboratoire MESuRS, Conservatoire National des Arts et Métiers, Paris, France
[2] Institut Pasteur, Conservatoire National des Arts et Métiers, Unité PACRI, Paris, France
[3] University Rennes, EHESP, REPERES (Recherche en pharmaco-épidémiologie et recours aux soins) – EA 7449, Rennes, France
[4] Centre régional de prévention des infections associées aux soins (CPias), Paris, France
[5] Sorbonne Université, INSERM, Institut Pierre Louis d'Epidémiologie et de Santé Publique, F75013, Paris, France
[6] University Versailles Saint Quentin en Yvelines, UMR 1181, « Biostatistics, Biomathematics, Pharmacoepidemiology and Infectious Diseases » (B2PHI), Saint Quentin en Yvelines, France
[7] Institut Pasteur, Pharmacoepidemiology and Infectious Diseases unit, Paris, France
[8] Inserm UMR 1181 (B2PHI), Paris, France

Supplement 1: results for the 2012 to 2015 period

Figure S1.1. Distribution of 1122 network distances for observed data and permutations for all episodes occurring between 2012 and 2015, France. The density distribution is shown for observed data network distances and the distances of permutations. Distances of zero correspond to paths that occur in the same county. Distances of one correspond to a range of distances between zero and one; the same applies to distances of 2-20.

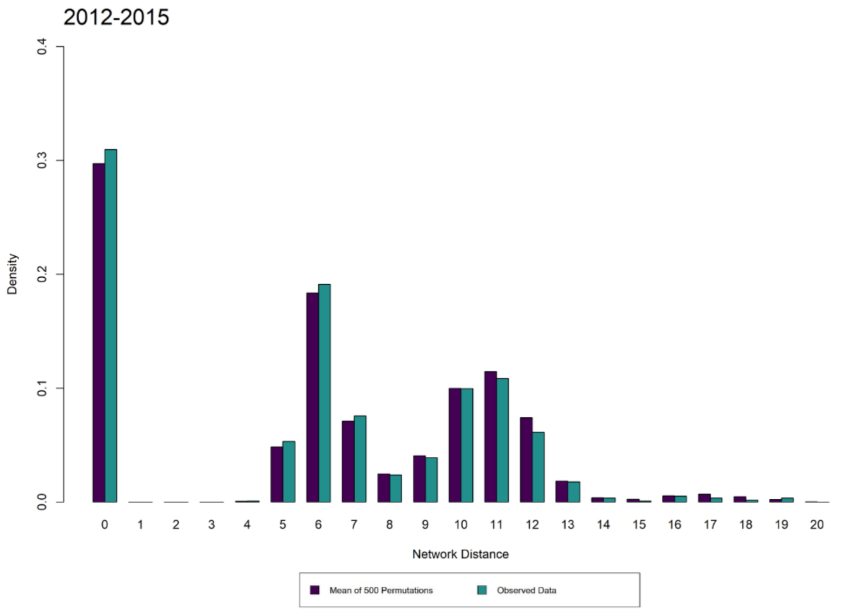

Figure S1.2. Sensitivity analysis on the impact of the time window chosen to select candidate transmitters. The distribution of the mean network distances between incident episodes and their potential infectors obtained for sliding 1-week time windows for all episodes occurring between 2012 and 2015. The mean network distance is plotted as a function of the first day of the 1-week time window, for observed data 1 and permuted data. For permutations, 95% confidence bands are provided.

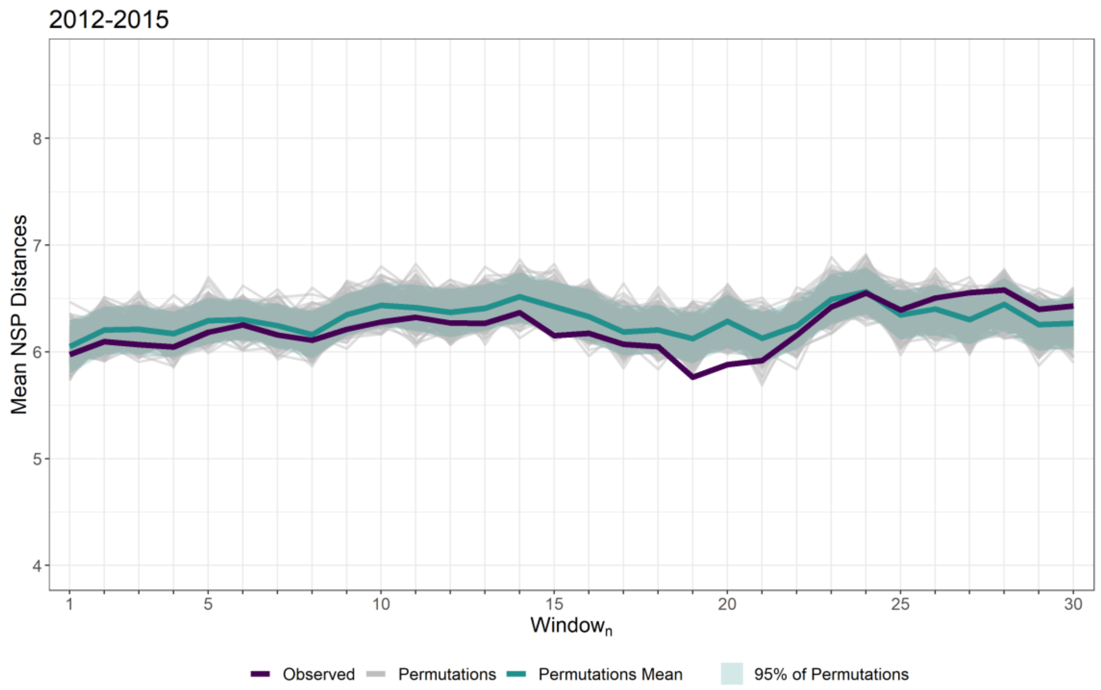

Supplement 2: spatial distances between potential infectors and their secondary cases

Table S2.1 Geographic distances to potential infectors. The mean distance in kilometers (km) between linked episodes occurring in all counties and among those in different counties.

Year	2012	2013	2014	2015
Mean geographic distances among all linked episodes with 95% CI	191 95% CI [145-237]	165 95% CI [134-197]	159 95% CI [135-183]	103 95% CI [89-117]
Mean geographic distances among linked episodes of different counties with 95% CI	232 95% CI [180-283]	225 95% CI [187-263]	222 95% CI [192-251]	163 95% CI [144-181]

Table S2.2 The proportion of geographic distances between for linked episodes.

Year	2012	2013	2014	2015
Proportion of episodes within (0,100] km	40%	39%	38%	47%
Proportion of episodes within (100,200] km	10%	17%	20%	21%
Proportion of episodes within (200,300] km	21%	15%	18%	15%
Proportion of episodes within (300,400] km	6%	10%	4%	9%
Proportion of episodes within (400,500] km	10%	6%	6%	3%
Proportion of episodes within (500,600] km	1%	2%	5%	2%
Proportion of episodes within (600,700] km	9%	9%	6%	3%
Proportion of episodes within (700,800] km	0%	1%	1%	0%
Proportion of episodes within (800,900] km	3%	1%	2%	1%

Supplement 3: sensitivity analysis

A sensitivity analysis was conducted in order to compare the baseline time window for selecting candidate transmitters to a sliding one-week window starting from 1 to 30 days ($W_{[1,8]}$ to $W_{[30,37]}$) prior to the incident episode starting date. The Wilcoxon paired-rank sum test p-values for all time windows shown in Figure 5 and Figure S1.2 are given in Table S3. When comparing all time windows for the combined years, the most significant difference was observed for $W_{[20,27]}$ and the baseline (Table S3). In 2012, only five windows showed a significant difference between the data and permutations. Conversely, the test showed a statistically significant difference between the mean network distances between incident CPE episodes and their potential infector for all windows in 2015. The windows with the lowest mean network distance corresponded to $W_{[3,10]}$ in 2015 and $W_{[19,26]}$ in 2014. The most significant differences were observed for $W_{[23,30]}$ in 2013, $W_{[19,26]}$ in 2014, and $W_{[20,27]}$ in 2015. However, for all time windows identifying potential infectors 20 to 30 days prior the incident episode date, the results were similar with significantly shorter distances for 2013-2015 but not for 2012.

Table S3. Sensitivity analysis. Mean network distances of observed data and 500 permutations: Wilcoxon paired rank sum test p-values for all time windows.

W_n	W_m	2012-2015			2012			2013			2014			2015		
		Mean distances		P-value	Mean distances		P-value	Mean distances		P-value	Mean distances		P-value	Mean distances		P-values
		O	P		O	P		O	P		O	P		O	P	
1	8	5.60††	5.72	2.51E-19***	7.15	7.27	0.25	6.23	6.15	0.20	5.97	6.05	1.31E-06***	4.86††	5.07	1.14E-14***
2	9	5.61	5.79	9.21E-22***	7.35	7.42	0.13	5.83†	5.88	1.98E-03**	6.10	6.21	2.83E-06***	4.90	5.17	2.39E-15***
3	10	5.59†	5.77	2.23E-27***	7.53	7.47	0.14	5.98	6.04	1.13E-04***	6.07	6.21	7.67E-08***	4.79†	5.06	3.48E-18***
4	11	5.64	5.79	1.52E-25***	7.46	7.41	0.45	6.13	6.06	6.11E-04***	6.05	6.17	1.79E-06***	4.87	5.15	6.46E-19***
5	12	5.70	5.87	3.78E-29***	7.25	7.27	0.25	6.27	6.29	2.13E-05***	6.18	6.29	2.64E-06***	4.97	5.27	5.05E-21***
6	13	5.76	5.91	1.51E-24***	7.07	7.11	0.05	6.26	6.36	3.21E-05***	6.25	6.30	1.65E-04***	5.06	5.32	8.31E-18***
7	14	5.75	5.86	2.56E-22***	6.88	6.85	0.11	6.51	6.56	5.53E-05***	6.16	6.25	1.29E-05***	5.03	5.21	2.86E-14***
8	15	5.75	5.86	3.54E-25***	6.93	7.05	3.78E-03**	6.61	6.72	2.10E-04***	6.11	6.16	3.56E-06***	4.97	5.15	4.78E-17***
9	16	5.86	5.96	1.26E-26***	6.86†	7.17	1.05E-03**	6.63	6.76	1.75E-04***	6.21	6.35	3.67E-08***	5.17	5.24	1.72E-15***
10	17	5.86	5.95	4.16E-21***	6.92††	7.17	3.02E-03**	6.45	6.52	1.34E-04***	6.28	6.44	1.09E-06***	5.21	5.25	3.38E-10***
11	18	5.89	5.97	2.23E-19***	7.11	7.43	0.01*	6.48	6.59	3.96E-05***	6.32	6.41	1.43E-05***	5.18	5.21	5.15E-10***
12	19	5.91	5.99	2.14E-18***	7.46	7.70	0.03*	6.36	6.45	1.68E-04***	6.27	6.37	2.44E-05***	5.19	5.25	1.66E-10***
13	20	5.91	6.02	3.33E-25***	7.27	7.64	1.99E-03**	6.38	6.48	3.20E-05***	6.27	6.41	2.95E-06***	5.24	5.29	1.98E-14***
14	21	5.94	6.02	3.05E-24***	6.94	7.25	0.01*	6.54	6.58	7.82E-05***	6.37	6.52	3.76E-06***	5.29	5.32	1.53E-14***
15	22	5.88	6.04	1.14E-26***	6.99	7.02	0.06	6.51	6.59	5.35E-05***	6.15	6.42	2.66E-08***	5.32	5.45	4.33E-16***
16	23	5.81	5.97	2.91E-23***	7.33	7.22	0.35	6.40	6.49	2.29E-05***	6.17	6.33	2.02E-06***	5.09	5.33	7.73E-15***
17	24	5.79	5.95	4.15E-23***	7.05	7.03	0.28	6.47	6.63	1.28E-05***	6.07	6.19	7.25E-07***	5.09	5.34	1.19E-13***
18	25	5.74	5.94	3.36E-24***	6.84	6.87	0.19	6.38	6.50	4.34E-04***	6.05	6.20	1.15E-06***	5.07	5.39	2.78E-17***
19	26	5.71	5.94	1.00E-25***	6.83	6.78	0.32	6.65	6.62	0.03*	5.76†	6.12	1.19E-08***	5.07	5.43	2.89E-20***
20	27	5.70	5.99	5.28E-29***	7.03	6.82	0.24	6.39	6.52	2.92E-04***	5.88††	6.29	1.91E-08***	5.09	5.53	5.52E-21***
21	28	5.61	5.89	2.71E-27***	7.34	7.24	0.14	6.15	6.32	1.07E-03**	5.92	6.13	9.78E-07***	4.86	5.35	7.17E-20***
22	29	5.71	5.93	5.90E-23***	7.31	7.19	0.11	6.02	6.31	1.47E-04***	6.16	6.24	1.54E-04***	4.96	5.33	8.20E-17***
23	30	5.87	6.08	4.86E-21***	7.16	6.99	0.21	5.98††	6.33	3.19E-06***	6.42	6.49	3.44E-03**	5.22	5.55	9.32E-16***
24	31	5.88	6.01	1.59E-19***	7.24	7.12	0.09	6.20	6.37	3.38E-05***	6.55	6.56	3.82E-03**	5.13	5.38	5.79E-13***
25	32	5.78	5.89	1.36E-21***	7.19	6.82	0.29	6.15	6.32	5.23E-05***	6.39	6.34	3.05E-03**	5.04	5.31	2.12E-15***
26	33	5.88	6.01	2.97E-23***	7.28	7.08	0.05*	6.25	6.44	2.19E-04***	6.51	6.40	0.01*	5.11	5.40	1.86E-17***
27	34	5.92	5.94	1.30E-13***	7.26	7.17	0.07	6.21	6.33	3.10E-03**	6.56	6.30	0.35	5.23	5.39	4.12E-11***
28	35	5.89	5.95	3.18E-16***	7.14	6.95	0.33	6.20	6.38	4.23E-05***	6.58	6.45	0.04*	5.22	5.41	3.25E-12***
29	36	5.86	5.86	8.90E-16***	7.29	7.04	0.89	6.20	6.26	4.28E-04***	6.40	6.25	4.17E-03**	5.23	5.34	2.39E-12***
30	37	6.04	6.00	1.85E-11***	7.57	7.32	0.60	6.38	6.38	4.61E-03**	6.43	6.27	0.02*	5.42	5.49	6.17E-10***

O: observed data; P: permutated data; * p-value < 0.05; ** p-value < 0.01; *** p-value < 0.001; † shortest significant observed network distance; †† second shortest significant observed network distance.
